# Improving ciaglia blue rhino technique for tracheostomy with a simple procedural modification

**DOI:** 10.1186/2197-425X-3-S1-A935

**Published:** 2015-10-01

**Authors:** F Imperatore, G Spinelli, F Imparato, PF Marsilia, L Mendetta, G Liguori, M De Cristofaro

**Affiliations:** Emergency Department Reanimation, A. Cardarelli Hospital, Naples, Italy; School of Anesthesia and Intensive Care, University of Naples 'Federico II', Naples, Italy

## Introduction

Percutaneous tracheostomy is one of the most commonly operative procedures performed in Intensive Care Unit (ICU) patients [1]. Ciaglia Blue Rhino technique (CBR) is used in our ICU. In our clinical practice we sometimes encountered difficulties in doing initial dilatation of the soft tissue and tracheal wall with the Blue Rhino dilator: it occurs expecially in young or obese patients and usually results in a longer procedure time and more related complications.Also other Authors report these difficulties [2].

## Objectives

Aim of this study is to present a simple procedural modification of Ciaglia Blue Rhino PDT in order to avoid the potential difficulty in carrying out initial dilation and greatly reduce the chance of seeing the posterior tracheal wall or create a false route during the dilation. After a preliminary experience with this new technique that showed its safety and in fact we replaced the standard CBR technique with the new one from January 2014.

## Patients and Methods

A retrospective cohort study in a medical/surgical ICU was carried out over a 24-month period: patient undergoing PDT between January 2014 to December 2014 (Group A: 70 patients) were retrospective analyzed and compared with patient who were undergoing PDT between January 2013 to December 2013 (Group B: 65 patients). In the Group A PDT was performed by using the modified CBR technique: the modification consist of a transverse cut (5 mm long and deep to the anterior tracheal wall) practiced, under guided light given by flexible bronchoscope trans illumination, before the trachea was punctured with a 14-gauge Teflon catheter introducer needle. In the group B we use the standard CBR technique [1]. In both groups we use fiber optic guide and Blue Rhino dilator (Cook Critical Care, Bloomington, IN, USA) was used to dilate the opening to a sufficient size.

## Results

One hundred and thirty five patients were entered into the study. Patient data and complications recorded are reported in table 1, 2. Differences among procedure time between the two groups was noted and summarized in table 3. Of note editing technique that we propose allows to reduce significantly the procedure time. Moreover it reduce the force required for the dilation of the tracheal wall without impacting on the effectiveness and safety of the original technique.

**Table 1:** Patient Characteristics.

	Group A	Group B	p value
male/female (%)	40 (57.1) / 30 (42.9)	36 (55.3) / 29 (49.7)	
age, years (range)	55.25 (18-88)	51.34 (20-86)	0,35
SAPSII	36.12 ± 11.15	37.10 ± 10.1	0,42
ICU admission patology	Trauma (16) Cerebral hemorrage (5) Cerebral ischemya (8) Abdominal surgery (11) Respiratory (18) Miscellaneous (12)	Trauma (15) Cerebral hemorrage (4) Cerebral ischemya (10) Abdominal surgery (7) Respiratory (17) Miscellaneous (10)

**Table 2:** Procedure related complications.

	Group A	Group B
Lowering of SaO2	0	0
Tracheal tear	0	3
Pneumotorax	0	0
Subcutaneous emphysema	0	0
Tracheal stenosis	0	0
Tracheo oesophagel fistula	0	1
Wound infection	1	0
Inability to complet procedure	0	0
Procedure mortality	0	0

**Table 3:** Procedure time.

	Group A	Group B	p-value
Mean ± SD(min)	4.7 ± 1.5	5.6 ± 3.1	< 0,001
Short, <5 min	50 (71,4%)	32 (49,2%)	
Moderate, 5-8 min	14 (20 %)	23 (35,4%)	
Prolonged, >8min	6 (8,6%)	10 (15,4%)	

## Conclusions

The simple modification we propose made the initial dilation easier to perform and prevented a prolonged procedure time. In addiction appears to reduce the incidence of complications such as lesions of the posterior tracheal wall and fractures of the tracheal rings.
